# Evaluation of Smile Aesthetics in Dental Students: Perceptions of Tooth Colour Changes Due to Incisor Inclination and Micro- and Mini-Aesthetic Characteristics Assessed by Professionals and Laypersons

**DOI:** 10.3390/dj13080380

**Published:** 2025-08-20

**Authors:** Eugen Bud, Alexandru Vlasa, Anamaria Bud, Mariana Pacurar, Sorana Maria Bucur, Daniela Esian, Elena Stepco, Olga Cheptanaru, Bianca Gabriela Nenec, Andrei Cosmin Nenec

**Affiliations:** 1 Department of Orthodontics, Faculty of Dental Medicine, George Emil Palade University Medicine, Pharmacy, Science, and Technology of Targu Mures, 38 Gh. Marinescu Str., 540139 Târgu Mureș, Romania; eugen.bud@umfst.ro (E.B.); mariana.pacurar@umfst.ro (M.P.); 2 Department of Periodontology and Oral-Dental Diagnosis, Faculty of Dental Medicine, George Emil Palade University of Medicine, Pharmacy, Science, and Technology of Targu Mures, 38 Gh. Marinescu Str., 540139 Târgu Mureș, Romania; 3 Department of Pedodontics, Faculty of Dental Medicine, George Emil Palade University of Medicine, Pharmacy, Science, and Technology of Targu Mures, 38 Gh. Marinescu Str., 540139 Târgu Mureș, Romania; anamaria.bud@umfst.ro (A.B.); daniela.esian@umfst.ro (D.E.); 4 Department of Dentristry, Faculty of Medicine, University Dimitrie Cantemir, 540545 Târgu Mureș, Romania; 5 “Ion Lupan” Department of Pediatric Oral-Maxillofacial Surgery and Pedodontics, Nicolae Testemitanu State University of Medicine and Pharmacy, 2004 Chișinău, Moldova; elena.stepco@usmf.md; 6 “Pavel Godoroja” Dental Propaedeutics Department, Nicolae Testemițanu State University of Medicine and Pharmacy, 2004 Chișinău, Moldova; olga.cheptanaru@usmf.md; 7 ENT Department, Târgu Mureș Emergency County Hospital, 540136 Târgu Mureș, Romania; bianca-gabriela.nenec@outlook.com; 8 Stomatology and Maxilo-Facial Surgery Department, Targu Mures Emergency County Hospital, 540136 Târgu Mureș, Romania; andrei_nenec@outlook.com

**Keywords:** colour perception, orthodontic torque, smile aesthetics, incisor inclination, CIELAB

## Abstract

**Background:** The present study investigated the relation between dental inclination, colorimetric variation, and aesthetic perception according to the modification of incisor inclination. Smile aesthetics, shaped by morphological factors and patient perception, are vital for social attractiveness and treatment success. This study aimed to assess the effect of varying head tilt on the perceived colour of upper central incisors by simulating changes in torque of the tooth, as well as evaluate factors influencing the perception of an aesthetic smile, including morphological characteristics and gingival aesthetic parameters. **Methods**: The study was comprised of three stages: colour analysis, evaluation of micro- and mini-aesthetic smile features, and an image-based assessment to determine evaluator perceptions and overall smile attractiveness. A sample of 50 students with complete, lesion-free anterior dentition was analysed. To simulate the effect of orthodontic torque changes during colour analysis, subjects tilted their heads downward and upward, representing palatal and buccal crown torque, respectively. Standardized macro-intraoral photographs were captured under controlled lighting conditions using a DSLR camera stabilized on a tripod in the different positions: the neutral head position (*p*0), 15° upward (*p* + 15), and 15° downward (*p* − 15). Digital colour analysis was conducted in the CIELAB colour space (L*, a*, b*). In the next stage, focusing on micro- and mini-aesthetic evaluation, an additional 50 smiles were generated using artificial intelligence via the SmileCloud program—one digitally enhanced smile per subject—complementing the initial set of 50 spontaneous smiles. These 100 smile images were evaluated by 50 laypersons and 50 dentists using a visual analogue scale via an online questionnaire, in order to assess perceptions, determine smile attractiveness, and quantify gingival aesthetic parameters. **Results**: The statistically significant regression results are as follows: those for the L* values in all three head inclinations: downward (−15 degrees), upward (+15 degrees), and total tilting (−15 to +15 degrees), as well as for the a* values for downward tilting and the b* values for total tilting. When the head is tilted downwards, the central incisors are positioned retrusively, and the L* b* values reveal a darker and more yellowish appearance, whereas, with the head tilted upwards, the central incisors protrude, and L* a* values indicate a brighter and more greenish appear. In the evaluation stage of the smile aesthetics study, no significant differences were observed in the judgments between laypersons and dentists or between males and females. Smiles with a high or average anterior line, parallel arc, upward lip curvature, visible first/second premolars, a smile index of 5.08–5.87, and symmetry score of 1.04 were rated as more attractive. Significant asymmetries were observed between upper dental hemi-quadrants in gingival contour and interdental papilla height, highlighting subtle morphological variations relevant to smile aesthetics. **Conclusions**: Aesthetic assessment revealed that the findings suggest a measurable impact of head position on dental colour perception and aesthetic evaluation. Evaluator variables including profession and gender exerted negligible effects on aesthetic perception, whereas smile attractiveness features and gingival aesthetic parameters demonstrate significant clinical applicability in patient management.

## 1. Introduction

The concept of beauty has captivated human interest since antiquity, and dentistry shares common principles with other aesthetic disciplines. With ongoing improvement in quality of life, aesthetic expectations have significantly increased, particularly concerning facial and dental appearance. A smile is a defining element of facial expression, inherently linked to both mastication and phonation [1,2]. Oral aesthetics play a key role in first impressions and social perception, with multiple studies indicating that an attractive smile positively influences how individuals are judged and treated [1,2]. Each individual develops a unique ideal of dental beauty, with satisfaction regarding one’s own smile closely associated with self-esteem and psychological traits [3]. Despite growing interest in aesthetic dental treatments, their necessity remains debatable, primarily due to ethical consideration [4]. Smile aesthetics depend on a balanced integration of dental and gingival features, including tooth size, shape, visibility, and colour [5,6]. Recent advancements in digital smile design (DSD) and digital planning tools have enhanced our understandings of how multiple variables, such as colour and inclination, interact to shape aesthetic outcomes [4]. These perceptions vary not only among individuals but also depending on the observer’s professional background: while dental professionals tend to identify minor discrepancies, laypersons are more tolerant of such variations unless they are particularly pronounced [7].

Recent studies have emphasized the clinical relevance of gingival contour analysis, highlighting key parameters such as interdental papilla height, gingival zenith position, gingival line direction, and overall symmetry. These gingival aesthetic factors, alongside the characteristics of healthy gingiva, are essential for accurate diagnosis, treatment planning, and the achievement of optimal outcomes in soft tissue rehabilitation [8,9,10,11,12,13]. Understanding these perceptual differences is essential for developing individualized treatment plans aligned with patient expectations, ultimately enhancing treatment satisfaction and outcomes [14].

Facial aesthetics during speech and smiling are critically determined by the interplay between the lips and teeth [15]. Proffit’s model categorizes aesthetic harmony into three domains: macro-aesthetics (overall facial proportions), mini-aesthetics (lip–tooth–smile relationship), and micro-aesthetics (tooth and gingival characteristics) [16,17]. Tooth colour is another critical determinant of dental aesthetics, often being the first attribute noticed by observers. Even a subtle variation in shade can significantly influence judgements of attractiveness [18]. Previous research has demonstrated that variations in tooth inclination can significantly alter perceived tooth colour from a fixed observer’s perspective. Buccal crown torque has been associated with a perceptibly lighter, redder, and bluer appearance, whereas palatal crown torque tends to produce a darker and redder visual effect [19]. Moreover, the integration between colour, shape, and alignment can either enhance or diminish the perceived harmony of a smile [20]. When combined with variations in teeth inclination, colour changes may either compensate for or accentuate perceived aesthetic disharmonies [21]. Among anterior teeth, the maxillary incisors play an essential role in determining the visual harmony of a smile [22]. The inclination, in particular, has been shown to significantly affect both facial aesthetic and the perception of dental attractiveness [13]. Despite a growing body of literature examining the impact of individual factors on dental aesthetics, few studies have investigated the interaction between them- specifically, how incisor inclination can influence the perception of teeth brightness or colour through changes in light reflection and shadowing [23,24]. Therefore, this study investigates the influence of head tilt on the perceived colour of the upper central incisors by simulating changes in tooth torque, a relevant factor in orthodontic and aesthetic dental assessments. In addition, the research addresses micro- and mini-aesthetic components of the smile, emphasizing the necessity of integrating these intraoral features with broader facial harmony to achieve a comprehensive and patient-centred evaluation of smile aesthetics.

## 2. Materials and Methods

This cross-sectional study was conducted between January and June 2024 in the Department of Orthodontics, UMFST “George Emil Palade” University of Medicine, Pharmacy, Science and Technology of Târgu Mureș. Ethical approval was obtained from the University’s Ethic Committee (Approval No. 2709/27.12.2023), and all participants provided informed consent.

A total number of 58 students were initially enrolled based on an a prior power analysis. The sample size, consisting of dental students, was calculated based on the statistical data described by Ciucchi, P. [19]. After applying the inclusion criteria, 50 subjects were found eligible. The study sample included 25 male and 25 female research participants, aged 20–30 years, with a mean age of 26.2.

The following inclusion criteria were used: age between 18 and 35 years old; full permanent dentition; and absence of anterior maxillary restorations, malformations, or periodontal disease.

The authors employed the following exclusion criteria for the study: active or prior orthodontic treatment, missing or supernumerary anterior teeth, caries or prosthetic restorations on anterior teeth, crowding of the upper incisors, smokers, oral-facial trauma, systemic disease, and congenital abnormalities. For the smile images assessment stage (via an online Google Forms questionnaire), 100 observers were included: 50 laypersons (25 males, 25 females) without formal education or professional experience in dentistry and 50 dentists (25 males, 25 females) with a minimum of 3 years of clinical dental practice.

Oral hygiene procedures, including professional brushing and air-polishing, were performed prior to imaging. Soft disposable retractors Optragate 3D (Ivoclar, Schaan, Liechtenstein) or sterilized unilateral–commissural retractors (Aksim Surgical Ltd, Middlesex, England) ensured unobstructed visualization of the maxillary anterior teeth. The retractor size was pre-selected to minimize distortion. Participants were instructed to maintain maximum intercuspation during the procedure. Excess saliva was removed without drying the teeth to prevent desiccation

**Figure 1:**
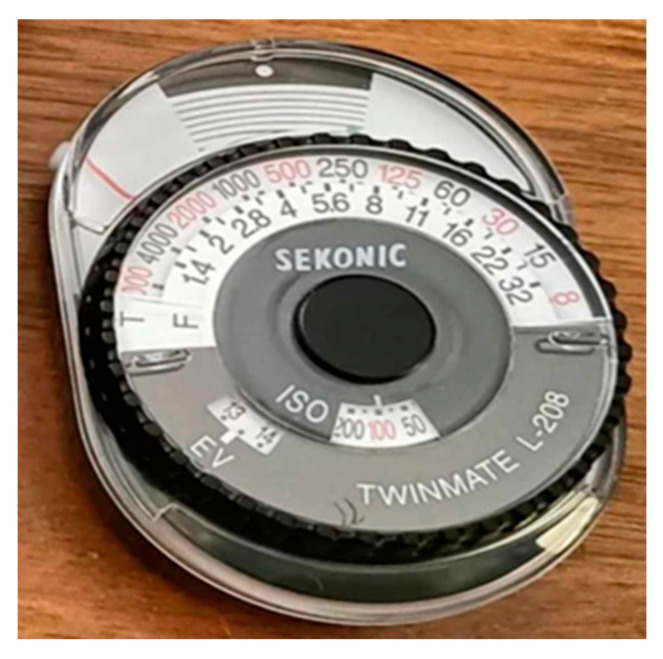
Photometer used during the study methodology (Sekonic, Tokyo, Japan).

Standardized photographic conditions were ensured by conducting all sessions in a windowless room. A rigid metal frame was assembled to support two 18 W circular, artificial D65-equivalent light sources (6500 K) (Heapol 18 W 1600 lm, Lohuis, Brasov, Romania) aimed frontally and superiorly at the subject’s face. This illumination was selected for its ability to closely replicate natural daylight conditions. The intensity level of the light directed at the subject was measured using a photometer (Sekonic, Tokyo, Japan), as seen in Figure 1, and any variation in output led to the replacement of the light source to ensure consistent illumination.

Polarization filters (K&F Concept, Shenzhen, China) were attached to the camera lens, and A4-sized polarizing sheets (Lacerta, Vienna, Austria) were affixed to the light sources to minimize glare and reflections caused by dental surface morphology.

Each research participant was seated on an identical chair positioned 10 cm from a neutral-coloured wall to avoid shadow artefacts. Head positioning followed natural head posture principles: subjects gaze into a mirror placed at eye level, establishing a horizontal Frankfort plane and bi-pupillary line. These anatomical landmarks were marked with a dermatographia pencil to define the reference position (*p*0). A customized angular measurement device was utilized to precisely guide and standardize participants’ head orientation during positioning.

**Figure 2:**
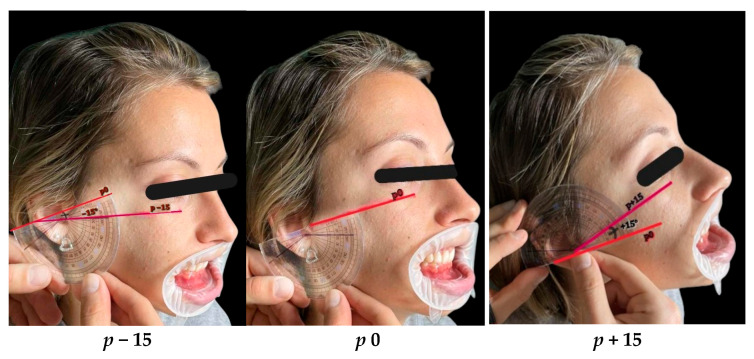
Schematic representation of head positioning based on the Frankfort horizontal plane, illustrating forward and backward rotational head inclination.

To stimulate orthodontic torque effects, participants tilted their heads downwards (*p* − 15°) and upwards (*p* + 15°) from the *p*0 reference using a goniometric protractor aligned with the Frankfort plane, as seen in Figure 2. Head tilts were rehearsed and standardised, ensuring subjects were able to maintain the angulation comfortably during imaging.

**Figure 3:**
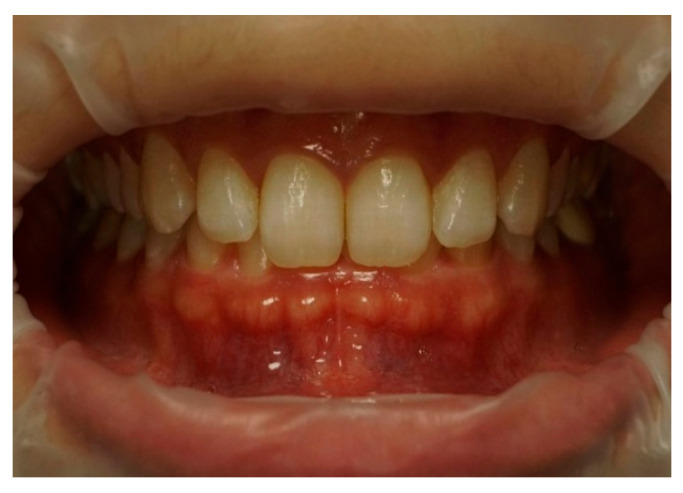
Intraoral photography corresponding to *p* − 15 head position for subject 1.

**Figure 4:**
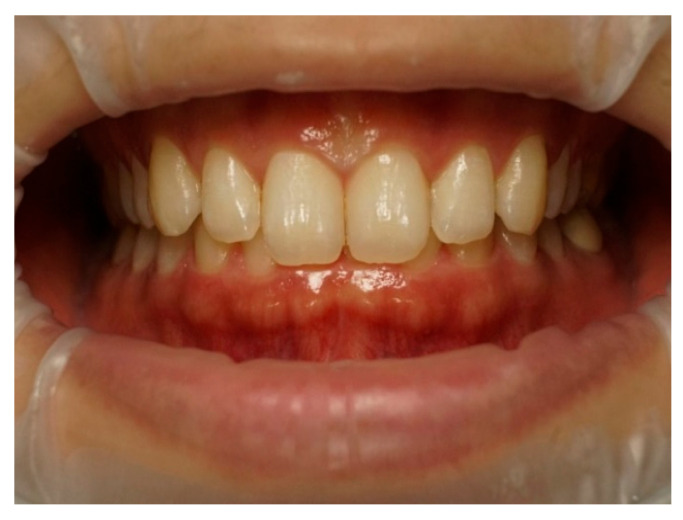
Intraoral photography corresponding to *p*0 head position for subject 1.

**Figure 5:**
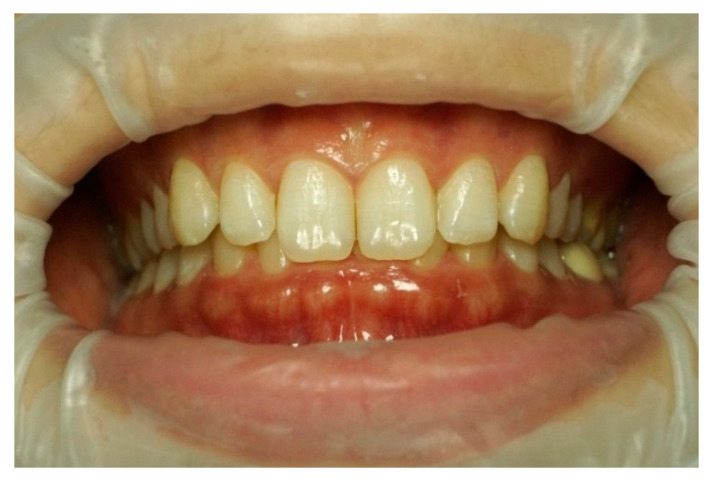
Intraoral photography corresponding to *p* + 15 head position for subject 1.

Frontal–intraoral macro photographs without flash were captured parallel to the Frankfort plane at the patient’s natural head position. The photographs were taken at three angles (+15°, 0°, and −15°) from a fixed distance of 0.75 m with a Sony α6000 DSLR camera (Sony Group Corporation, Tokyo, Japan) stabilized using a “Manfrotto” tripod (Lino Manfrotto + Co. S.p.A., Veneto, Italy) with a three-dimensional rotation head, flatness graduations, and height adjustment. The DSLR camera was set to manual mode with ISO 800, focal length f = 10, and shutter speed 1/125 s consistently applied across all images. A 70 mm macro lens (Ø 49 mm) was employed in conjunction with a set of three extension tubes measuring 16 mm, 10 mm, and 21 mm (Kooka, Shenzhen, China) to facilitate fine-tuning of the focal distance and achieve optimal sharpness. Photographs were saved in RAW format on a professional UHS SDXC memory card to ensure high-quality, uncompressed, and unprocessed files. Intraoral images were captured to document three distinct head tilt positions to achieve maxillary incisor inclinations of −15° (as seen in Figure 3), 0° (as seen in Figure 4), and +15° (as seen in Figure 5).

For the acquisition of extraoral facial photographs intended for smile design processing in the SmileCloud version 0.2.0 software, a digital camera equipped with an integrated CanonPowerShot G7X Mark II lens (Canon Inc, Ōta, Tokyo, Japan) was utilized, ensuring high-resolution image capture suitable for aesthetic calibration.

**Figure 6:**
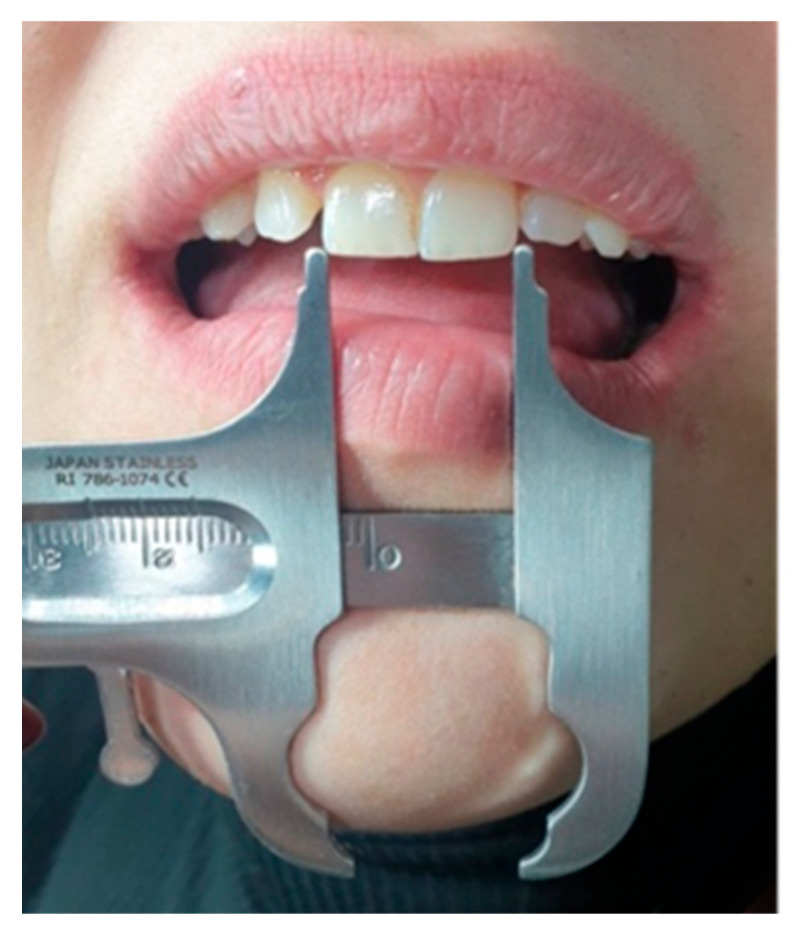
Measurements of M-D ⌀ at the level of the incisal embrasure.

These extra-oral images and images from the digital smile design (DSD) stage were processed using Adobe Photoshop version 25.4 and SmileCloud 1.194.2 version software to assess changes in tooth colour and aesthetic parameters. For image calibration and accurate linear measurements within the SmileCloud software, the mesiodistal width of each subject’s maxillary central incisor was physically measured at the incisal embrasure level using a Boley gauge. This anatomical reference was used in SmileCloud’s “Ruler” tool by digitally marking the mesial and distal contact points on the corresponding clinical photograph and inputting the measured value in millimetres, allowing the software to proportionally scale the image for precise digital analysis and smile design planning, as presented in Figure 6. This reference benchmark allowed accurate scaling of digital simulations generated through the AI-assisted SmileCloud platform.

**Figure 7:**
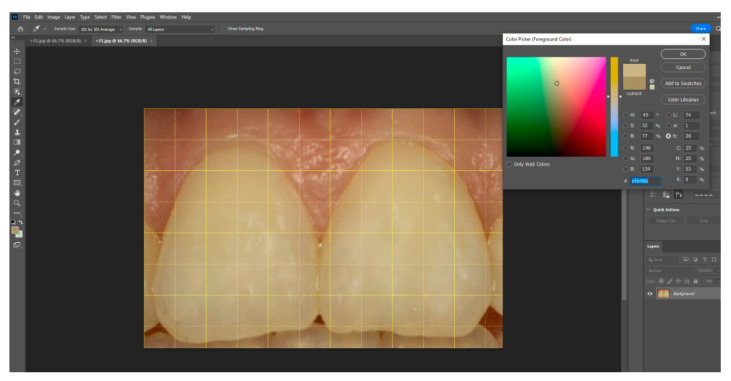
Image of the central maxillary incisors corresponding to the position *p* + 15 of the head inclination.

**Figure 8:**
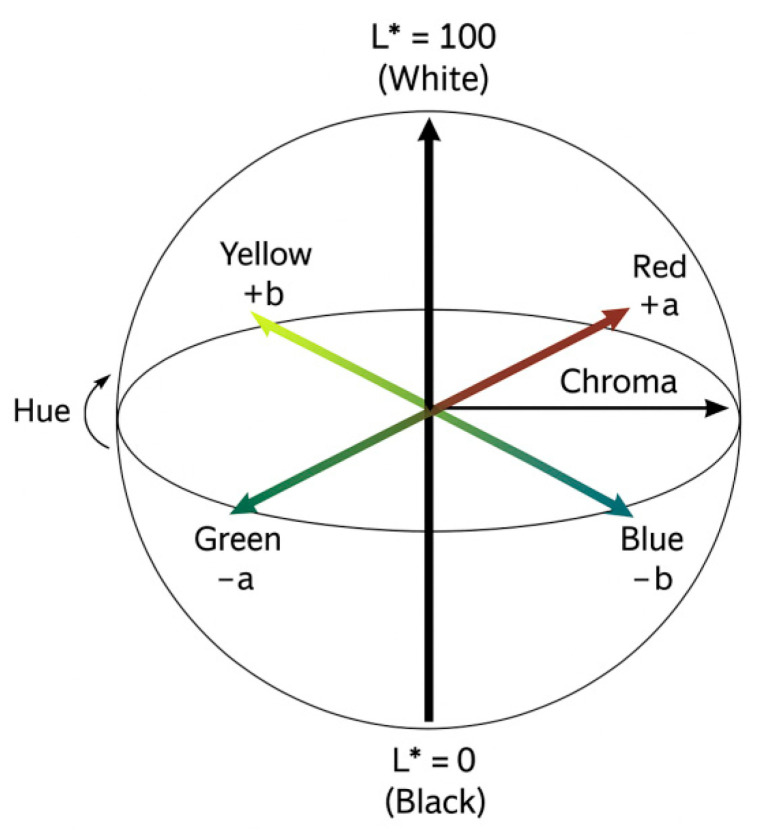
Schematic representation of CIE Lab*.

Using Adobe Photoshop, each image was cropped to isolate the central incisors. A pixel grid (101 × 101 px) was applied at the centre of the facial surface, and colour was sampled using the “colour sample” and “colour picker” tools (as shown in Figure 7). Colour values were expressed in the CIE Lab* colour space, L* represents lightness on a scale from 0 (black) to 100 (white), while a* and b* define chromaticity along the red–green and yellow–blue axes, respectively, each ranging from −100 to +100 (as seen in Figure 8) This model is grounded in the opponent-process theory of colour vision, which posits that red and green, as well as blue and yellow, are perceptually antagonistic and cannot be simultaneously present, allowing for colour description using single, continuous values on each axis. Data were tabulated in Microsoft Excel 2301 version for statistical analysis, categorized by incisor angulation. Mean CIE Lab* values for the central incisors were computed for each of the three head inclinations for each research participant. Paired *t*-tests assessed differences between positions (α = 0.05). Intra-rater reliability was evaluated on a random subsample (*n* = 10) using the intraclass correlation coefficient (ICC), with an ICC of 0.87 indicating excellent repeatability.

**Figure 9:**
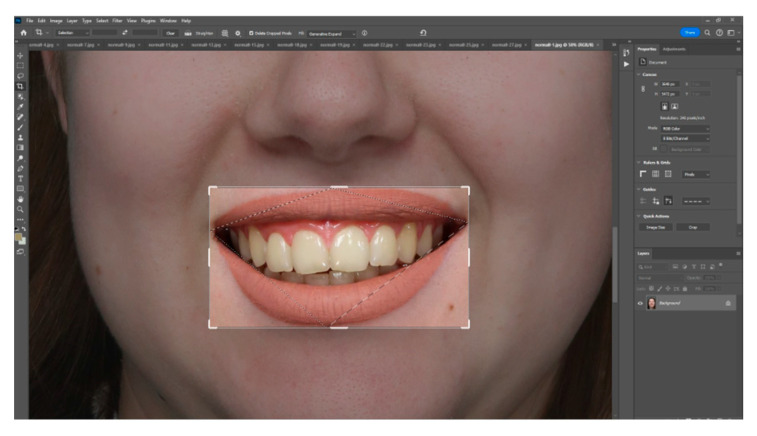
Representation of the smile region in Photoshop.

**Figure 10:**
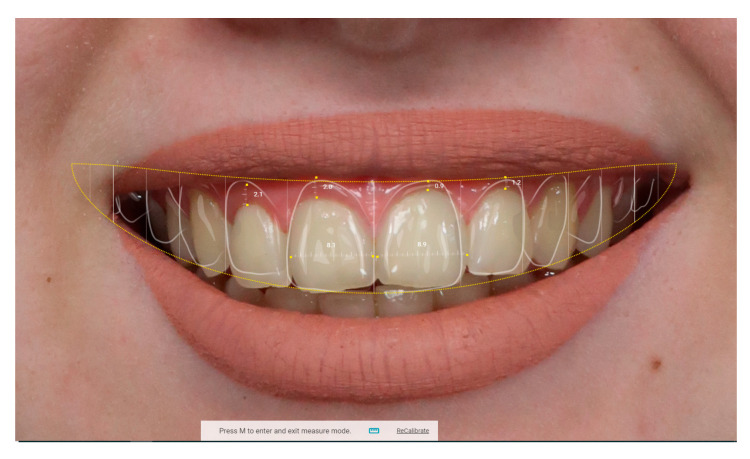
Digital mock-up and restorative space in SmileCloud.

In the second phase, smiles were digitally reconstructed in SmileCloud. The lower third of the face was cropped to define the smile region (bounded by superior and inferior labial points and bilateral cheilions) (as seen in Figure 9). Virtual tooth libraries were applied, and smile simulations were generated based on calibrated facial proportions. Key digital design steps included the following: facial segmentation, lip contour mapping, restorative space adjustment, and correlation between digital and physical dimensions using the SmileCloud “Ruler” function (Figure 10).

**Figure 11:**
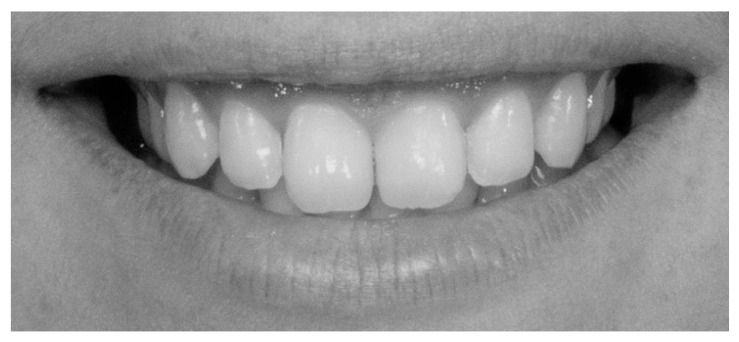
The unaltered, natural smile of a subject.

**Figure 12:**
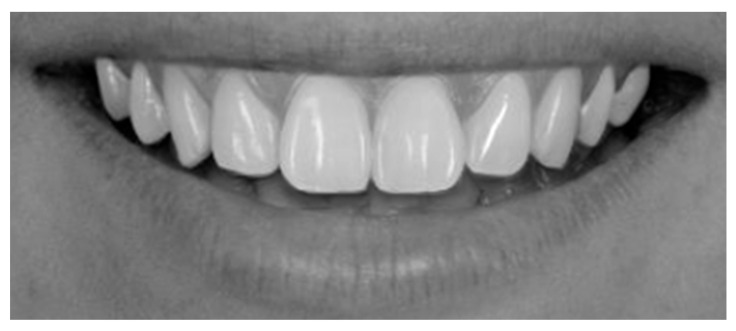
Digitally adjusted wider smile in SmileCloud, showing increased exposure of dental unites for enhanced aesthetic evaluation.

To augment the sample and facilitate comparative aesthetic analysis, 50 digitally optimized smile images were generated using the SmileCloud platform’s AI-driven smile design function. Each subject’s natural smile was processed to create a harmonized version, yielding a dataset of 100 standardized frontal smile images (50 unaltered and 50 AI-enhanced) for subsequent aesthetic evaluation. All images were transferred to a computer and anonymized using coded identifiers in Windows 10, version 22H2 (Microsoft). To minimize bias from extraneous facial morphology and skin tone, photographs were converted to 70 dpi grayscale JPEG format (576 × 288 pixels, 0% saturation) (Figure 11 and Figure 12).

Following the smile image assessment phase, during which 100 observers (50 dental professionals and 50 laypersons) rated smile attractiveness using a 10-point Visual Analogue Scale (VAS) administered via a standardized online questionnaire (Google Forms), the upper and lower quartiles (25th and 75th percentiles) of VAS scores were extracted. These subsets of the most and least attractive smiles (*n* = 50) were subsequently subjected to detailed morphological evaluation based on mini- and micro-aesthetic parameters.

Mini-aesthetic parameters were quantitatively evaluated using Adobe Photoshop CS6 and SmileCloud software. All measurements were performed based on a calibrated image enlargement ratio to ensure consistency and accuracy across the dataset. Six variables were assessed:

(1) Smile line, classified according to Liebert and Deruelle [25] as very high (>2 mm marginal gingiva visible), high (0–2 mm), average (gingival embrasure only), or low (no gingiva visible);
(2) Smile arch, categorized as parallel, straight, or inverted [26];
(3) Oral corridor width, categorized as wide, average, narrow, or minimal;
(4) Smile width, defined by the most posterior visible tooth (canine, first premolar, second premolar, or first molar) [27];
(5) Smile index, calculated as the ratio of intercommissural width (points 1–2) to interlabial gap (points 3–4);
(6) Dynamic smile symmetry, calculated using the formula [(1 − 3) + (1 − 4)]/[(2 − 3) + (2−4)].

**Figure 13:**
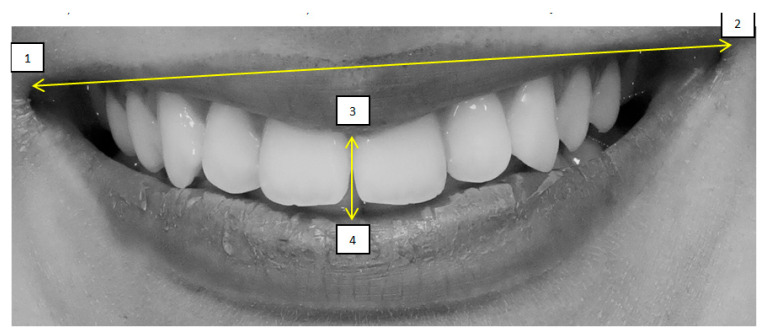
Smile index = $\frac{\left(1-2\right)}{\left(3-4\right)}$ = 4.65. (1 − 2) = Width (intercommisural width on smiling); (3 − 4) = Height (interlabial gap on smiling).

**Figure 14:**
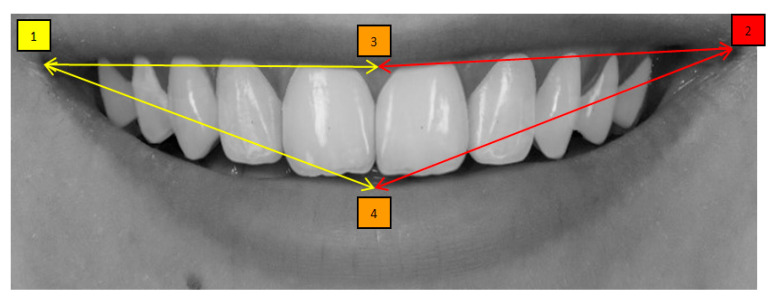
Dynamic smile symmetry = $\frac{\left(1-4\right)+(1-3)}{\left(2-4\right)+(2-3)}$ = 0.946. Point 1 corresponds to the right outer commissure; Point 2 to the left outer commissure; Point 3 to the midpoint of the inferior border of the upper lip; and Point 4 to the midpoint of the superior border of the lower lip.

The four anatomical reference points used for these calculations were: point 1 (right labial commissure), point 2 (left labial commissure), point 3 (midpoint of the inferior border of the upper lip), and point 4 (midpoint of the superior border of the lower lip)—Figure 13 and Figure 14.

**Figure 15:**
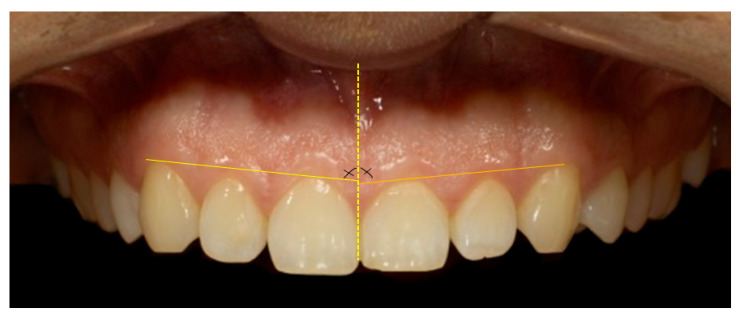
Gingival line angle (GLA): 89.24 in quadrant 1; 87.33 in quadrant 2. Yellow vertical line represents the facial midline, while the horizontal yellow lines indicate the respective gingival line angles. The cross angle marks the anatomical reference point corresponding to the intersection of the midline with the gingival zenith of the maxillary central incisors.

**Figure 16:**
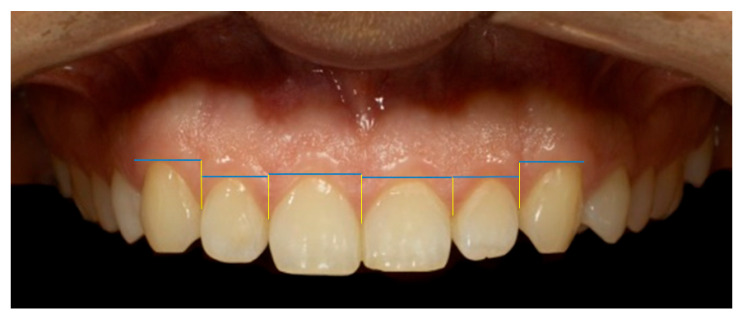
Micro-aesthetic parameter: inter-dental papilla height (IPH). The blue horizontal lines indicate the reference gingival margin levels, while the yellow vertical lines represent the measurement points of papillary height. The cross (×) marks the anatomical reference point corresponding to the midline between the maxillary central incisors, used as a landmark for measurement standardization.

Micro-aesthetic parameters (gingival aesthetic) assessed included the gingival line angle (GLA)—assessed as the angulation between the gingival contour and the maxillary midline—as shown in Figure 15 [28]. Additionally, the mesial and distal interdental papilla height (IPH) was measured for the anterior maxillary dentition—specifically, the central incisors, lateral incisors, and canines. IPH was defined as the vertical distance from the GZ of the corresponding tooth to the apex of the adjacent interdental papilla—Figure 16 [29].

**Figure 17:**
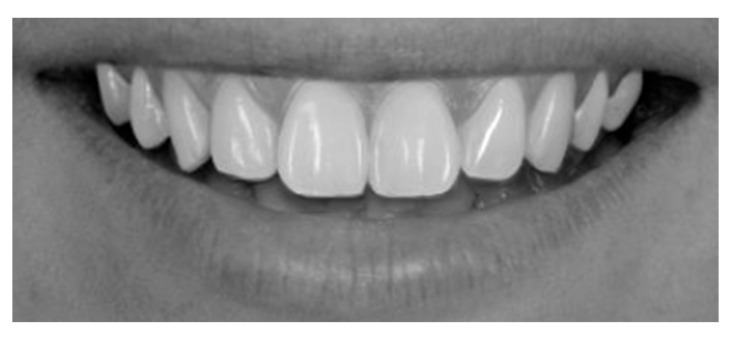
Parallel smile arch.

**Figure 18:**
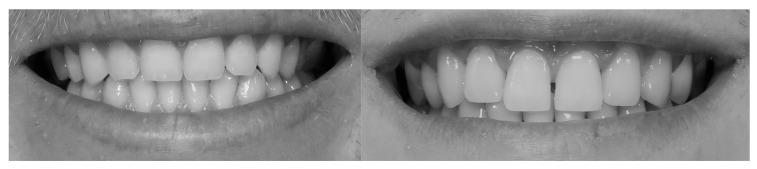
Straight smile arch.

Frontal view of a posed smile demonstrating the display of maxillary anterior teeth and gingival architecture, Figure 17 demonstrates a parallel smile arc, whereas Figure 18 illustrate a straight smile arc; both images were converted to grayscale to facilitate online aesthetic evaluation.

Statistical analyses were performed using Origin 2024b (v10.15) and IBM SPSS Statistics v29. Based on smile attractiveness scores, two primary groups were defined: Group A (attractive smiles) and Group B (unattractive smiles). Each group was further subdivided by gender (male/female) and professional background (dental/non-dental). Quantitative data were tested for normality using Kolmogorov–Smirnov and Shapiro–Wilk tests. For normally distributed data, parametric tests (unpaired *t*-tests) were used. Categorical variables were assessed using Chi-square (*χ*^2^) and Wilcoxon Signed Rank tests. A significance level of *p* < 0.05 was applied throughout.

## 3. Results

**Figure 19:**
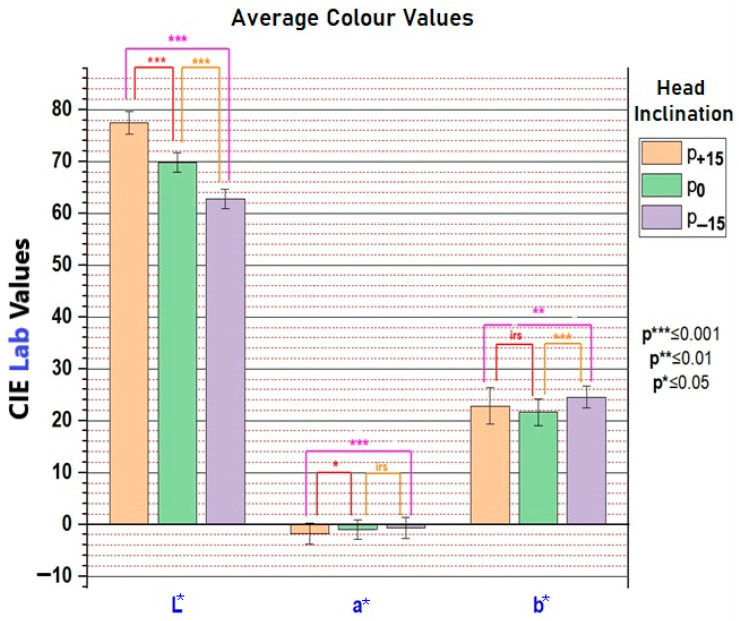
Average values of dental colour parameters (L*, a*, b*) for entire group of subjects. IRS indicates an irrelevant statistic.

Colorimetric analysis revealed the following: L* values (brightness) were significantly different across all inclinations (*p* < 0.001); a* values showed significant change only at +15° inclination; b* values were significantly different at both +15° and −15° inclinations. The segments in the graph in Figure 19 represent standard deviations. Statistical significance was assessed using the *t*-test: *p* * and *p* ** indicate results obtained at different sensitivity thresholds.

Overall, upward head inclination relative to the natural head position resulted in an apparent proclination of the maxillary incisors, which was associated with higher lightness values and perceptibly lighter tooth coloration. In contrast, downward head inclination produced an apparent retroclination of the incisors, correlating with reduced lightness values and a darker colour appearance. These findings indicate a consistent variation in perceived tooth colour across different head tilts due to simulated torque changes.

**Figure 20:**
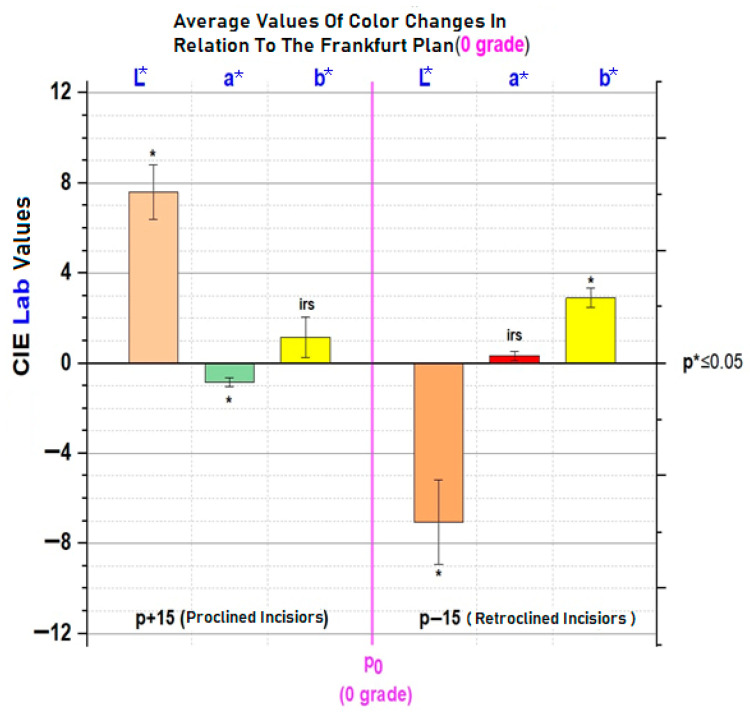
Colour changes of incisors for each colorimetric parameter (L*, a*, b*) at two different inclinations (*p* + 15°, *p* − 15°) relative to the reference position (*p*0). The segments in the chart indicate the standard deviations; irs = statistically irrelevant.

Colour differences in a* (green–red axis) and b* (blue–yellow axis) were also documented. At *p* − 15, the increase in b* indicates a more yellow hue, while at *p* + 15, the decrease in a* suggests a greener (less red) hue. These shifts support the rejection of the null hypothesis that head inclination does not influence perceived tooth colour, as seen in Figure 20.

The intraclass correlation coefficients (ICCs) for intra-rater reliability among the four randomly selected evaluators were 0.568, 0.673, 0.753, and 0.742, respectively, indicating acceptable reliability for the Visual Analogue Scale (VAS) assessments.

Comparative analysis of smile aesthetic variables—including anterior smile line, smile arch, size of the oral corridor, width of smile, smile index, and dynamic smile symmetry—between dentists and laypersons (Table 1), as well as between male and female evaluators (Table 2), revealed no statistically significant differences within the attractive smile subgroups. These findings suggest that, despite professional background or gender, observer perception of key smile characteristics remains consistent across groups.

A higher frequency of images displaying high or average anterior smile lines and parallel smile arcs was observed in the attractive smile subgroup compared to the unattractive subgroup. Conversely, low or very high smile lines and straight or inverted smile arcs were significantly less prevalent in the attractive subgroup. These differences were statistically significant. The distribution patterns of these four features were consistent across both evaluator groups (dentists and laypersons). Additionally, quantitative analysis revealed that both the smile index and dynamic smile symmetry values were significantly greater in the attractive subgroup, indicating a correlation between these parameters and perceived smile attractiveness (Table 3).

Thirty full-smile photographs—evaluated as attractive by both laypersons and dental professionals—were selected for detailed analysis of gingival aesthetic variables. The sample included 13 male and 17 female subjects. Quantitative assessments of gingival parameters are presented in Table 3. Statistically significant differences were found between the right and left sides in several gingival aesthetic characteristics, indicating measurable asymmetry even within objectively attractive smiles.

## 4. Discussion

This study demonstrated that changes in head inclination significantly influences the colorimetric perception of the maxillary central incisors. By simulating orthodontic torque through anterior and posterior head tilts, we were able to quantify alterations in colour parameters and associate them with perceived attractiveness. The L* value (lightness) showed consistent and statistically significant variation with head movement: anterior tilting (*p* − 15) resulted in darker teeth, while posterior tilting (*p* + 15) resulted in brighter teeth. This aligns with optical principles, as posterior tilt increases surface exposure to direct lighting, enhancing reflection and perceived brightness. Notably, a reduction in L* from 70 to 63 with anterior tilt may simulate the natural darkening of teeth due to aging, as previously reported in studies that examined age-related changes over 10 years [30].

The Frankfort horizontal plane (FHP) was used as the reference for determining head position due to its long-standing acceptance as a reproducible anatomical baseline in both dentistry and craniofacial research [31]. Defined by a line passing through the porion (upper margin of the external auditory meatus) and the orbitale (lowest point on the inferior margin of the orbit), the FHP closely approximates a natural head position when individuals look straight ahead at eye level [32]. This anatomical reference is widely employed because it offers standardization across studies and enables accurate comparisons of craniofacial and dental parameters across individuals and populations [33]. According to Solow and Tallgren (1971) [34], the Frankfort plane aligns closely with the natural head position in a relaxed, upright posture and is considered reliable for photogrammetric and cephalometric analyses.

The findings align partially with previous work by Cucchi and Killaridis [19], who found significant differences in L* and a*, but not b*, during anterior inclination. Our study, in contrast, revealed significant variation in b* rather than a* under similar conditions. These discrepancies may be attributed to differences in lighting source characteristics, enamel/dentine translucency, and the angle of light incidence.

No individual measurement of tooth inclination in the Frankfort horizontal position was conducted for the patients in this study, as the focus was not on precise angular dental measurements but rather on simulating the visual impact of typical orthodontic torque movements through changes in head position. The head tilt of ±15 degrees was selected based on prior literature [19,31,33], which demonstrated that such angular changes in head posture correspond geometrically to clinically realistic torque variations of the maxillary incisors seen in orthodontic practice, particularly during treatment of Class II Division 2 malocclusions. This magnitude of inclination has also been shown to produce visually perceptible differences in tooth appearance, including shade perception, making it a valid choice for testing the hypothesis [34].

The aesthetic evaluation supports these physical measurements. A total of 95% of observers selected brighter incisors (*p*0 or *p* + 15), underscoring the impact of perceived brightness on smile attractiveness. This preference remained consistent across genders and professions, though dentists were more critical of inverted smile arches and wide oral corridors, an agreement with previous findings by Al-Johany et al. [35].

Analysis of mini-aesthetic parameters revealed statistically significant asymmetry in interdental papilla height and gingival line angle between the upper quadrants. These differences, although subtle, may influence perception of smile balance and merit consideration in clinical assessments. Micro-aesthetic indices such as smile index and dynamic symmetry were significantly correlated with attractiveness, confirming previous research on the diagnostic and perceptual value of these metrics [13,36].

The findings indicate that high and average smile lines were most commonly associated with attractive smiles across both genders. Conversely, very high and low smile lines predominated among unattractive smiles. These results support previous literature suggesting that both excessive gingival display (gummy smile) and limited incisal display may negatively influence aesthetic perception [37,38].

Similarly, a parallel smile arch was found to be significantly more common among attractive smiles, while straight and inverted arches were more often associated with unattractive smiles. The smile arch contributes to the harmonization of the dental curvature with the contour of the lower lip, and deviations from this alignment may result in reduced visual harmony [39].

The oral corridor was narrower or minimal in attractive smiles. In contrast, unattractive smiles frequently showed wide corridors, which may create a “dark space” effect and disrupt the continuity of dental display. These findings are consistent with prior research emphasizing that excessive buccal corridors negatively influence smile aesthetics, particularly in frontal views [40,41].

Regarding smile width, attractive smiles were most often limited to the first or second premolars, while unattractive smiles frequently extended to the molars. This suggests that excessive exposure of posterior teeth may detract from smile harmony, possibly due to an imbalance in proportion and spatial composition [42].

The smile index and dynamic smile symmetry were both significantly higher for attractive smiles compared to unattractive ones. These quantitative indicators offer objective tools to assess smile aesthetics, aligning with the current trend toward evidence-based aesthetic evaluations in dentistry. The smile index, which reflects the ratio of smile width to height, and dynamic symmetry, which measures the balance between the right and left sides during smiling, appear to be critical determinants in aesthetic perception [43].

Across professional groups, smiles with a high or average smile line and a parallel arch were deemed the most aesthetic. This aligns with previous findings that moderate gingival display and congruence between dental curvature and lip contour are aesthetically preferred [27,44,45].

Participants across all domains associated minimal to narrow oral corridors and moderate smile widths (premolar extent) with increased attractiveness, while wide oral corridors and molar-level widths were more frequent in unattractive smiles. These features likely disrupt the proportional balance of the smile and introduce visual “voids”, which have been reported to decrease attractiveness [41].

Statistically significant differences in smile index and dynamic smile symmetry between attractive and unattractive smile groups across all professional fields highlight the objectivity and universality of these parameters in smile analysis. These results validate the use of quantitative smile metrics in clinical diagnostics and aesthetic planning [43,46].

Future research directions may include larger and more demographically diverse populations to enhance the generalizability of the findings. It is recommended to standardize viewing conditions during aesthetic assessments and incorporate patients with malocclusions, such as Angle Class II/2 to evaluate pre- and post-treatment changes in both dental colour and perceived smile attractiveness. Additionally, longitudinal studies involving orthodontic torque application are necessary to clarify the interactions between initial incisor inclination, facial posture, and aesthetic perception. Integration of cephalometric analysis may improve the accuracy of torque simulation models and provide a more robust framework for individualized treatment planning.

However, the following limitation must be acknowledged: the study sample consisted solely of dental students, which may limit generalizability. Additionally, visual assessment using varied digital screens could induce inconsistency due to lighting conditions, screen resolution, or observer fatigue.

## 5. Conclusions

This study highlights the significant influence of head inclination on the colorimetric perception of maxillary central incisors. A posterior head tilt (*p* + 15°) results in incisors that appear brighter and greener, while an anterior tilt (*p* − 15°) renders the teeth darker and more yellowish. These perceptual variations were statistically significant for the L* parameter across all positions, for a* at posterior tilt, and for b* at both anterior and posterior inclinations. The comprehensive evaluation of smile characteristics and gingival aesthetic parameters contributes valuable reference data to the field of dental aesthetics. These findings may serve as evidence-based aesthetic benchmarks to guide clinical decision-making in restorative, orthodontic, and periodontal treatment planning.

## Figures and Tables

**Table 1:** Comparison of mini-aesthetic smile characteristics split by profession.

Characteristics of Smile	Classification	Attractive Smiles			Unattractive Smiles		
		Dentists *p* (%)	Laypersons *p* (%)	*p* *	Dentists *p* (%)	Laypersons *p* (%)	*p* *
Smile line	Very high	0 (0%)	2 (8%)	0.255	11 (44%)	11 (44%)	1
	High	10 (40%)	13 (52%)		1 (4%)	1 (4%)	
	Average	14 (56%)	10 (40%)		3 (12%)	3 (12%)	
	Low	1 (4%)	0 (0%)		10 (40%)	10 (40%)	
Smile arch	Parallel	21 (84%)	22 (88%)	0.683	4 (16%)	7 (28%)	0.492
	Straight	4 (16%)	3 (12%)		12 (48%)	12 (48%)	
	Inverted	0 (0%)	0 (0%)		9 (36%)	6 (24%)	
Size of the oral corridor	Wide	0 (0%)	0 (0%)	0.089	15 (60%)	14 (56%)	0.478
	Average	2 (8%)	2 (8%)		5 (20%)	4 (16%)	
	Narrow	13 (52%)	14 (56%)		5 (20%)	7 (28%)	
	Minimal	10 (40%)	9 (36%)		0 (0%)	0 (0%)	
Width of smile	Canine	0 (0%)	0 (0%)	0.124	6 (24%)	5 (20%)	0.689
	First premolar	8 (32%)	8 (32%)		11 (44%)	12 (48%)	
	Second premolar	10 (40%)	11 (44%)		3 (12%)	1 (4%)	
	Molar	7(28%)	6 (24%)		5 (20%)	7 (28%)	
		Media ± SD		*p* **	Media ± SD		*p* **
Smile index		5.46 ± 0.38	5.50 ± 0.36	<0.001	4.34 ± 0.43	4.31 ± 0.43	<0.001
Dynamic smile symmetry		1.03 ± 0.05	1.10 ± 0.08	<0.001	0.89 ± 0.03	0.90 ± 0.03	<0.001

*p* * values calculated using the Chi-square test. *p* ** values calculated using the Independent Samples *t*-test.

**Table 2:** Comparison of mini-aesthetic smile characteristics split by gender.

Characteristics of Smile	Classification	Attractive Smiles			Unattractive Smiles		
		Men *p* (%)	Women *p* (%)	*p* *	Men *p* (%)	Women *p* (%)	*p* *
Smile line	Very high	2 (8%)	2 (8%)	0.366	11 (44%)	10 (40%)	0.736
	High	11 (44%)	13 (52%)		1 (4%)	1 (4%)	
	Average	11 (44%)	9 (36%)		3 (12%)	6 (24%)	
	Low	1 (4%)	1 (4%)		10 (40%)	8 (32%)	
Smile arch	Parallel	21 (84%)	19 (76%)	0.479	8 (32%)	9 (36%)	0.117
	Straight	4 (16%)	6 (24%)		9 (36%)	8 (32%)	
	Inverted	0 (0%)	0 (0%)		8 (32%)	8 (32%)	
Size of the oral corridor	Wide	0 (0%)	0 (0%)	0.355	13 (52%)	15 (60%)	0.352
	Average	3 (12%)	4 (16%)		8 (32%)	7 (28%)	
	Narrow	14 (56%)	12 (48%)		4 (16%)	3 (12%)	
	Minimal	8 (32%)	9 (36%)		0 (0%)	0 (0%)	
Width of smile	Canine	6 (24%)	5 (20%)	0.371	2 (8%)	2 (8%)	0.583
	First premolar	9 (36%)	8 (32%)		7 (28%)	5 (20%)	
	Second premolar	8 (32%)	10 (40%)		7 (28%)	9 (36%)	
	First molar	2 (8%)	2 (8%)		9 (36%)	9 (36%)	
		Average ± SD		*p* **	Average ± SD		*p* **
Smile index		5.49 ± 0.38	5.49 ± 0.38	<0.001	4.34 ± 0.44	4.29 ± 0.48	<0.001
Dynamic smile symmetry		1.03 ± 0.05	1.06 ± 0.05	<0.001	0.89 ± 0.03	0.84 ± 0.05	<0.001

*p* * values calculated using the Chi-square test. *p* ** values calculated using the Independent Samples *t*-test.

**Table 3:** Comparison of the components of the micro-aesthetics of the smile between the two upper dental hemi-quadrants; GALº= the angle of the aesthetic gingival line; HPI = the height of the interdental papilla; SD= the standard deviation; *p* * = *t*-test.

	Quadrant 1 (Average ± SD)	Quadrant 2 (Average ± SD)	*p* *
GALº	89.22 ± 3.02	85.44 ± 4.48	<0.001
HPI central incisor (mesial)	4.206 ± 0.103	4.186 ± 0.157	<0.001
HPI central incisor (distal)	4.114 ± 0.114	4.152 ± 0.115	<0.001
HPI lateral incisor (mesial)	3.68 ± 0.127	3.717 ± 0.1	<0.001
HPI lateral incisor (distal)	3.435 ± 0.104	3.476 ± 0.1	<0.001
HPI canine (mesial)	4.094 ± 0.127	4.166 ± 0.097	<0.001
HPI canine (distal)	3.551 ± 0.046	3.518 ± 0.119	<0.001

## Data Availability

The raw data supporting the conclusions of this article will be made available by the authors on request.
